# Use of emergency contraception among women with experience of domestic violence and abuse: a systematic review

**DOI:** 10.1186/s12905-018-0652-7

**Published:** 2018-09-25

**Authors:** Natalia V. Lewis, Theresa H. M. Moore, Gene S. Feder, John Macleod, Penny Whiting

**Affiliations:** 1 Centre for Academic Primary Care, Population Health Sciences, Bristol Medical School, University of Bristol, 39 Whatley Road, Bristol, BS8 2PS UK; 2 Centre for Primary Care and Public Health, Blizard Institute, Barts and the London School of Medicine and Dentistry, Queen Mary University of London, 58 Turner Street, London, E1 2AB UK; 3 NIHR CLAHRC West, Whitefriars, Lewins Mead, Bristol BS1 2NT and Bristol Medical School, University of Bristol, 39 Whatley Road, Bristol, BS8 2PS UK

**Keywords:** Domestic violence and abuse, Intimate partner violence, Emergency contraception, Hormonal contraception, Systematic review

## Abstract

**Background:**

Exposure to domestic violence and abuse (DVA) results in a reduction of women’s use of regular contraceptives. This evidence suggests that women exposed to DVA are more likely to have unprotected sexual intercourse and therefore may use more emergency contraception (EC) than those women who are not exposed to DVA. We aimed to test this hypothesis through evaluating the evidence for an association between exposure to DVA and use of EC.

**Methods:**

We systematically searched eight electronic databases from inception until December 2017, checked references and citations, and contacted corresponding authors. Primary studies that evaluated the association between exposure to DVA and use of EC were included. Two reviewers were involved in screening, data extraction, quality assessment and analysis. We evaluated the quality of included studies with the adapted Newcastle-Ottawa Scale. We used tables and descriptive text to summarise and synthesise the data. Odds ratios (ORs) and 95% confidence intervals (CIs) for each estimate of the association between DVA and use of EC were plotted on a forest plot.

**Results:**

Our search retrieved 1216 records of which six studies with 15,297 women were included. Five studies were observational; one study included intervention on the outcome (advance supply of EC). All studies were at high risk of bias. Four studies provided evidence of an association between DVA and EC use – ORs from 1.51 (95% CI 1.13, 2.02) to 6.50 (95% CI 4.15, 10.17). Two studies found no evidence of a such association – ORs 0.46 (95% CI 0.11, 1.96) and 0.76 (95% CI 0.29, 1.98). The latter differed by how the authors recruited participants, measured EC use and adjusted for confounders.

**Conclusions:**

This systematic review provides some evidence of increased use of EC among women exposed to DVA. Request for EC can indicate possible exposure to DVA. Therefore, each consultation for EC could be an appropriate context for clinical enquiry about DVA and signposting/referral to specialist DVA services.

**Protocol registration:**

PROSPERO CRD42017058221.

**Electronic supplementary material:**

The online version of this article (10.1186/s12905-018-0652-7) contains supplementary material, which is available to authorized users.

## Background

Domestic violence and abuse (DVA) against women is a worldwide human rights, public health and clinical problem [1]. The UK government defines DVA as “any incident or pattern of incidents of controlling, coercive, threatening behaviour, violence or abuse between those aged 16 or over who are, or have been, intimate partners or family members regardless of gender or sexuality. The abuse can encompass, but is not limited to: psychological, physical, sexual, financial, emotional” [2]. The World Health Organisation (WHO) estimates that approximately a third of ever-partnered women have experienced lifetime physical and/or sexual violence by an intimate partner or sexual violence by a non-partner [3]. Health care providers are often women’s first point of professional contact because most women attend health services at some point, especially sexual and reproductive health services. The WHO and NICE guidelines recommend a case-finding (synonym clinical enquiry) approach to identifying patients with experience of DVA: healthcare providers asking those women who present with clinical associations of DVA about safety in their relationship and at home, and signposting or referring those who disclosed to specialist DVA services [4–6]. Therefore, professional awareness of clinical associations of DVA is a crucial first step towards health-care response to DVA.

DVA results in significant morbidity and disability among women, with the biggest impact on their mental and reproductive health [1] including an increased risk of unintended pregnancy and abortion [7, 8]. One of the proposed mechanisms linking DVA and unintended pregnancy is reproductive coercion (RC), when males control the contraceptive use and pregnancy outcomes of their female partners [9, 10]. Recent systematic reviews found that exposure to DVA resulted in a reduction in condom and oral contraceptive use [11, 12]. This finding suggests that women exposed to DVA are more likely to have unprotected sexual intercourse, and therefore may need more emergency contraception (EC), than those women who are not exposed to DVA. EC with oral (hormonal contraceptive pills levonorgestrel, or ulipristal acetate) or intrauterine (copper intrauterine device) method is an evidence-based intervention for preventing unintended pregnancy [13, 14]. However, the recent reviews [11, 12] included only one study that had EC use as an outcome [15]. The objective of this systematic review was to evaluate the evidence for an association between exposure to DVA and use of EC.

## Methods

We followed the Centre for Reviews and Dissemination [16] and Cochrane guidance for undertaking [17] and reporting [18] systematic reviews in health care [see PRISMA checklist in Additional file 1]. The protocol was registered on PROSPERO (CRD42017058221).

### Search strategy

MEDLINE, EMBASE and PsycINFO on OVID; CINAHL on EBSCOhost, The Cochrane Library and Web of Science were searched from inception to December 2017. We used terms for DVA and combined these with terms for EC using the Boolean operator ‘AND’. In addition, we searched for grey literature on Google (first 6 pages), Opengrey.eu, Clinical trial registers, websites of NHS Choices, Department of Health, relevant medical and pharmacy associations, and charities in the field of reproductive health and DVA. No language or publication restrictions were applied [Additional file 2]. Identified references were downloaded into Endnote X7 software and deduplicated. To find additional studies, one reviewer examined reference lists of the included papers, checked their citations through the Web of Science and contacted all corresponding authors.

### Study selection

Primary studies of any design that evaluated the association between exposure to DVA (any measure) and use of EC were eligible for inclusion. Systematic reviews that met this criterion were included if searches were conducted within the past year. Studies that exclusively enrolled pregnant women were excluded. We also excluded studies that only considered RC without measuring DVA because the two phenomena can occur independently [10].

Endnote references were imported into an MS Access database developed for this study. Two reviewers independently screened titles and abstracts and full-text papers against the inclusion criteria. Any discrepancies between reviewers were resolved though discussion and consensus. All papers excluded at the full-text screening stage were documented along with the reasons for exclusion.

### Data extraction

We developed data extraction forms in MS Word and piloted and refined these on the first three papers. To minimise bias and errors, one reviewer extracted the data and a second reviewer checked the extraction in detail. Disagreements were resolved through discussion or referral to a third reviewer. We extracted information on study characteristics (author, publication year, country, design, setting), population (age, race/ethnicity, relationship status), measures of DVA and EC, cofounders, and data on the association between DVA and EC. Where both adjusted and crude estimates were reported, we extracted adjusted estimates. Where studies only reported frequencies, we calculated crude odds ratios.

### Quality assessment

We adapted the Newcastle-Ottawa Scale (NOS) [19] to assess the quality of included studies. We distinguished between applicability and risk of bias. To assess applicability, we evaluated whether the exposed group was representative of a general population of women of reproductive age [20] and of a country population of women at risk of exposure to DVA [3]. Risk of bias was assessed for the following domains:

I. Selection of the non-exposed group. Selection of exposed and non-exposed groups from the same population indicated low risk of bias.
II. Ascertainment of exposure. Measurement of DVA through diaries or regular interviews using validated questionnaire indicated low risk of bias.
III. Comparability of groups. Adjustment for core cofounders (age, socioeconomic status, race/ethnicity) and use of other reversible contraceptive methods indicated low risk of bias [11, 12].
IV. Assessment of outcome. Measurement of EC use through diaries or regular interviews and ascertainment of the temporal relationship between the exposure and outcome indicated low risk of bias.

Although NOS allocates ‘stars’ for adequate methods we chose not to adopt this approach due to evidence suggesting that numerical quality ratings are not helpful in differentiating between studies of high and low risk of bias [21]. We replaced the ‘star’ system with signalling questions, factual questions that flag the potential for bias, for each domain leading to an overall domain risk of bias rating.

### Analysis

Due to differences in how DVA and EC use were defined and measured in included studies, it was not possible to conduct a meta-analysis. We used tables and descriptive text to summarise the data and discuss differences in results between studies. We plotted ORs and 95% confidence intervals for each estimate of the association between DVA and EC use on a forest plot, stratified by study. Where studies reported adjusted ORs, these were included in the plot in preference to crude ORs. We used Stata 10 to produce the forest plot.

## Results

**Fig. 1.**
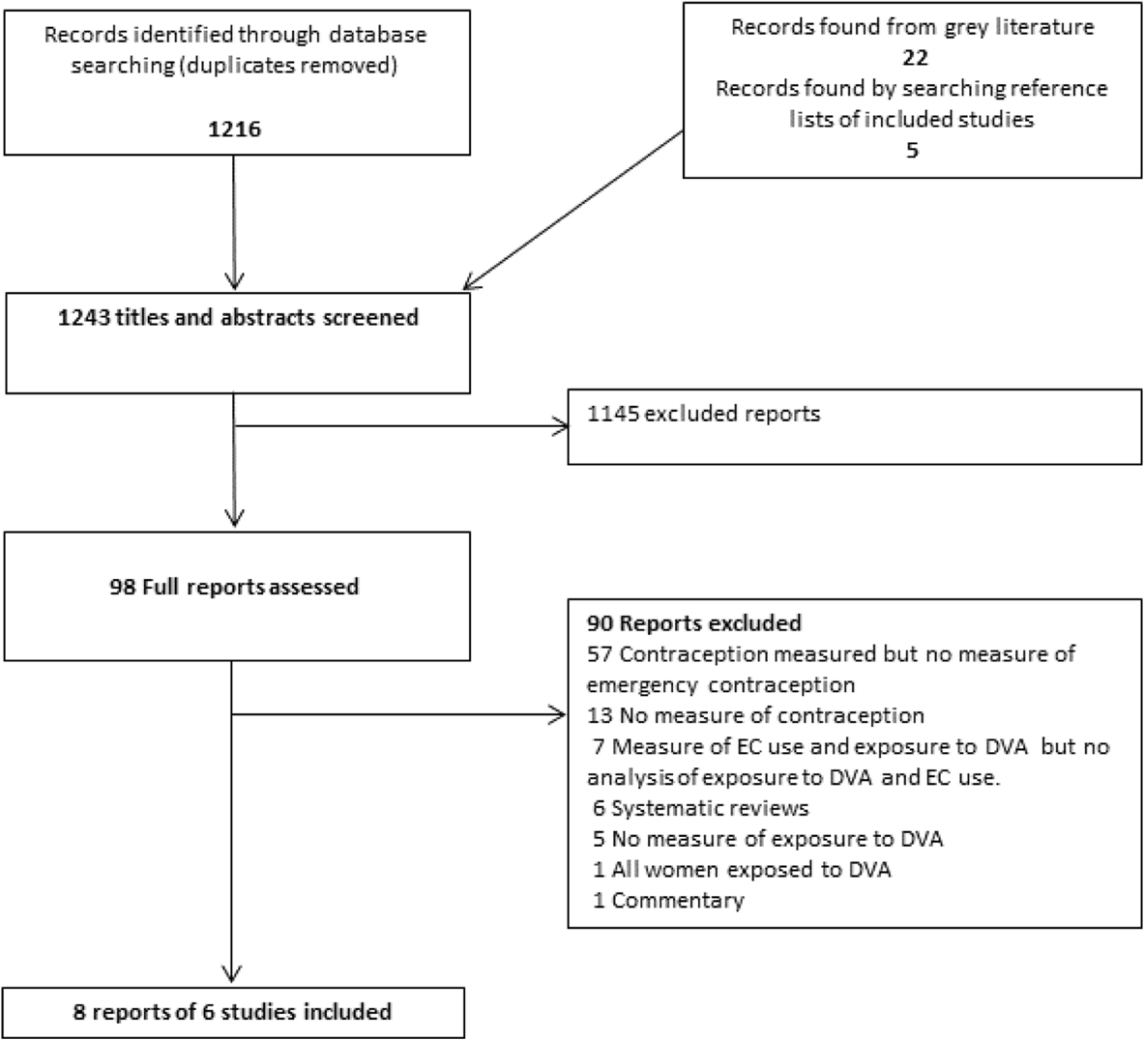
Flow of studies through the review

Our search retrieved 1216 records of which six studies (eight reports) were included (Fig. 1) [15, 22–27]. One study was reported in three publications [24, 27, 28]. Excluded studies are listed in Additional file 3 with reasons for exclusion.

### Study characteristics

**Fig. 2:**
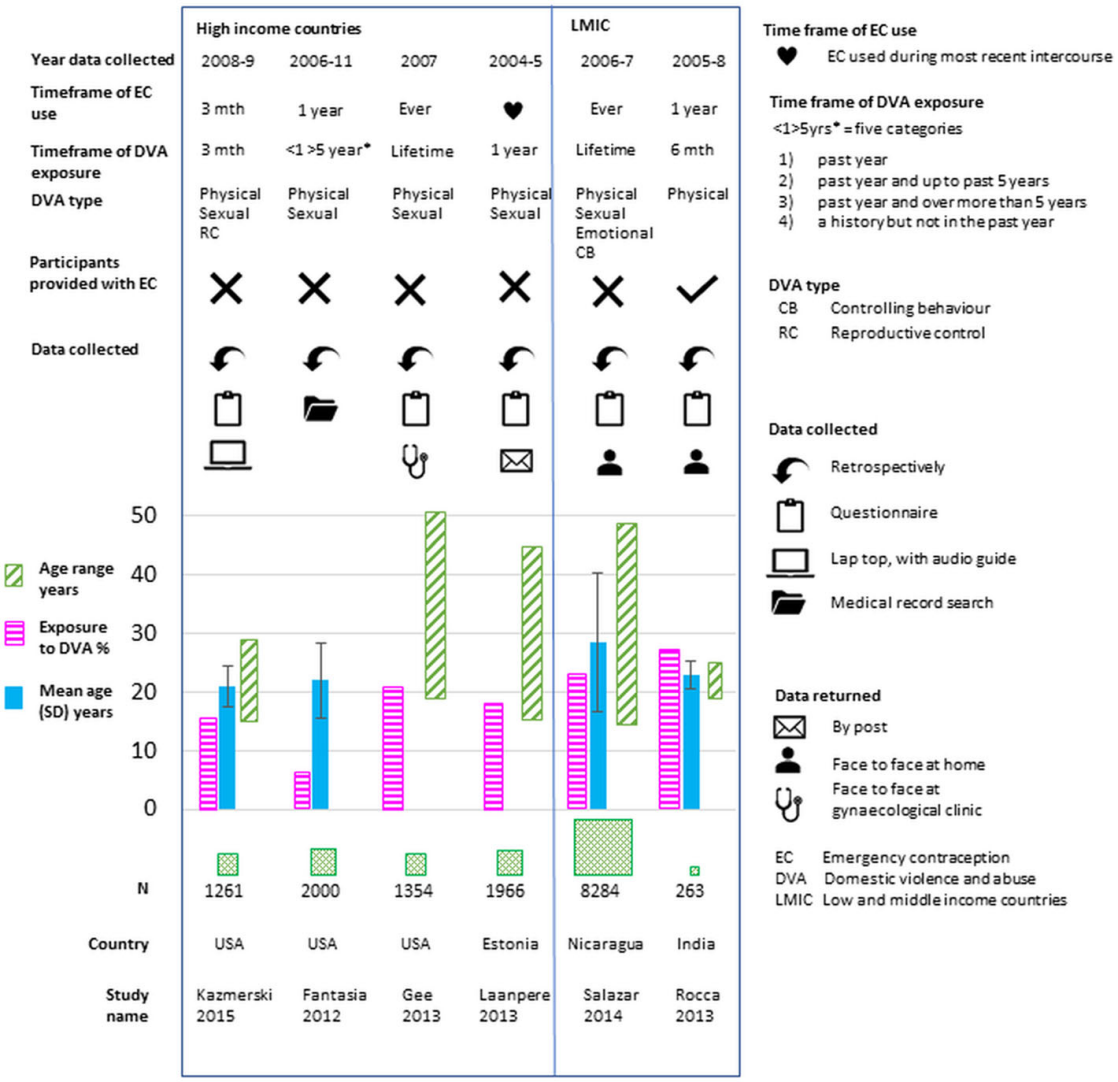
Studies characteristics

Studies were conducted in the USA [15, 22, 25], Estonia [24, 27], Nicaragua [23] and India [26] between 2012 and 2015 and involved 15,297 women. Figure 2 summarises characteristics of the included studies. An additional table file describes study characteristics in detail [see Additional file 4]. All studies were cross sectional observations, except for Rocca et al. [26] which included intervention on the outcome – women were recruited, offered advance supply of EC pills and, those who accepted the supply, were followed up for 1 year. This study sample was also highly selective – most women who declined the advance supply opted out because of their husbands’ disapproval of EC. Across studies, samples were drawn from general [23, 24, 26] and clinical populations [15, 22, 25]. Data were collected through self-administered questionnaires [22, 24, 25], face-to-face interviews [23, 26], and examination of medical records [15]. Most of the women were at the younger end of the reproductive age range [20], although the study from Estonia included older women [24].

The prevalence of DVA ranged from 4% [24] to 54% [23], although the type and timeframe of the exposure varied considerably. The exposure was measured with self-developed questions [15, 24, 26] and standardised validated questionnaires [22, 23, 25]. The studies measured different types of DVA – from physical only [26], through physical/ sexual [22, 24], to physical/ sexual/ emotional [15, 25]. One study measured RC alongside physical/sexual DVA [22]. The timeframe of exposure ranged from within the past 3 months [22] to lifetime [23].

Four studies measured use of oral EC [23, 25, 26, 28], while two studies did not explicitly state which method of EC was assessed [15, 22]. Use of EC was assessed on a continuum from “following the most recent sexual intercourse” [24] to “lifetime” [23]. Only one study measured the frequency of EC use [22].

### Quality assessment

Only the Nicaraguan study was applicable to both the general population of women of reproductive age and country DVA population [23] (Table 1). Other studies were considered at high concern for applicability as included women were at the younger end of the reproductive age range [22, 26] and of lower socioeconomic status compared to a country DVA population [15, 25]. All six studies were at high risk of bias on at least one domain.

**Table 1:** Applicability and risk of bias assessed with the adapted Newcastle Ottawa Scale

Study first author, year	Fantasia 2012	Gee 2013	Laanpere 2013	Rocca 2013	Salazar 2014	Kazmerski 2015
Applicability of study sample						
1. Representative of the general population of women of reproductive age * Is the study sample representative of the female population of reproductive age?*	No	No	No	No	Yes	No
2. Representative of the general DVA population * Is the study sample truly representative of the coutry DVA population?*	No	Yes	No	No	Yes	No
Risk of bias						
I. Selection of the non-exposed group * Is the non-exposed group drawn from the same population as the exposed group?*	Low	Low	Low	Low	Low	Low
II. Ascertainment of exposure * Was the ascertainment of exposure prospective?* * Was the exposure measured with a valid methos of assessment?*	High	High	High	High	High	High
III. Comparability of groups * Did study control for age, socio-economic status, race/ethnicity, use of other reversible contraception methods?*	High	Low	High	High	High	High
IV. Assessment of outcome * Was the ascertainment of outcome prospective?* * Was the outcome ascertained with a valid methos of assessment?* * Was the temporality of the outcome* vs *exposure assessed?*	High	High	High	High	High	High
Overall risk of bias for study	High	High	High	High	High	High

Quality appraisal tool is the Newcastle-Ottawa Scale [19] adapted for this study. DVA domestic violence and abuse. Overall risk of bias for study is a reflection of the least favourable assessment for a single domain - e.g. if one domain is high risk whole study is high risk. Signalling questions that flag the potential for bias are shown in *italic*: Answer 'Yes' to signaling question indicates high risk of bias, answer 'No' indicates low risk of bias

### Association of DVA with EC use

**Fig. 3:**
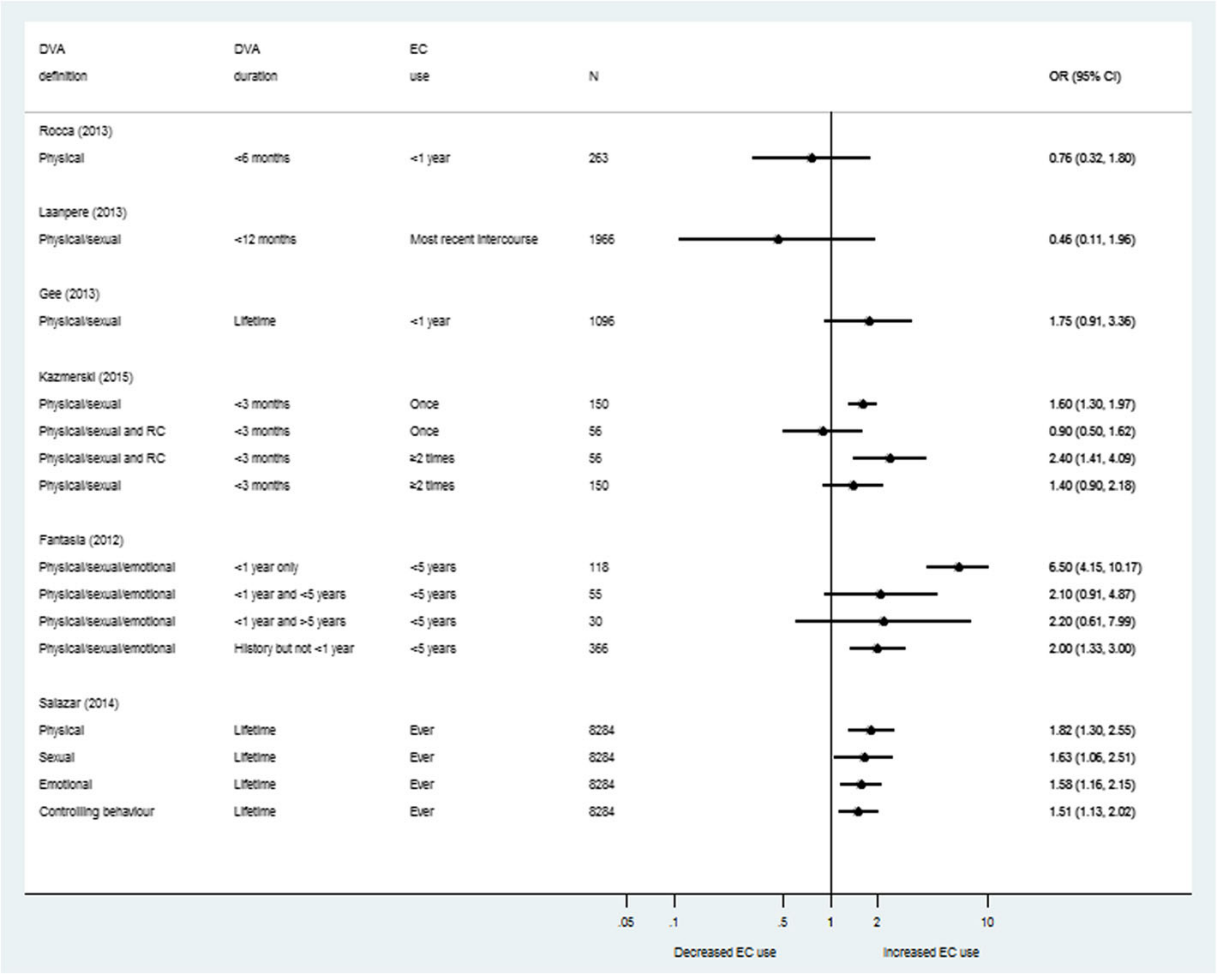
Forest plots: Odds ratios and 95% confidence intervals for use of emergency contraception among those exposed compared to those not exposed to domestic violence and abuse

Four studies provided evidence of an association between DVA and EC use (Fig. 3). The largest Nicaraguan study by Salazar et al. (*n* = 8234) [23] found strong evidence of an association for all types of DVA with no evidence of a difference between them – ORs ranged from 1.51 (95% CI 1.13, 2.02) to 1.82 (95% CI 1.30, 2.55). The American study by Fantasia et al. [15] explored associations between DVA exposure over four time-periods and EC use in the past year. This study found the strongest association for EC use and DVA in the past year (OR 6.50, 95% CI 4.15, 10.17). Exposure in the past year and extending beyond this to the past five or more years or historical DVA showed weaker associations (OR 2.00, 95% CI 1.33, 3.00 to 2.20, 95% CI 0.61, 7.99). Kazmerski et al. [22] categorised exposure as DVA alone, or DVA + RC, and measured EC use as “once”, or “more than once”, over the past 3 months. For women who had experienced DVA + RC, there was no association between exposure and use of EC once (OR 0.90, 95% CI 0.50, 1.62), but a strong association between exposure and use of EC more than once (OR 2.40, 95% CI 1.41, 4.09) [22]. Gee et al. [25] evaluated lifetime exposure to DVA and EC use within the past year and found weak evidence of an association (OR 1.75, 95% CI 0.91, 3.36).

Two studies found no evidence of an association between DVA and use of EC [24, 26]. The Estonian study by Laanpere et al. [24] was the only one that did not report adjusted ORs. This study also measured EC use at one time point, while other studies assessed outcome over a specific time period. The Indian study by Rocca et al. [26] was based on a highly self-selected sample and had an intervention on the outcome – advance supply of EC.

## Discussion

### Main findings

This systematic review of six studies with 15,297 women provides some evidence of increased use of EC among women exposed to DVA. The evidence is applicable to younger women in high- and middle-income countries. Four studies suggested that women who have experienced DVA are more likely to use EC compared to other women, while two studies did not find such a link. Null findings in the latter can be explained by the differences in how the authors recruited participants, measured EC use, and dealt with confounders. The included studies had high concerns regarding applicability and were at high risk of bias for ascertainment of exposure, comparability of exposed and unexposed groups, and assessment of outcome.

Our findings are in line with a recent meta-analysis which showed a causal relationship between exposure to DVA and reduction in use of regular contraception methods [11]. Our findings support previous research linking DVA and unprotected intercourse through RC [10, 29]. The Kazmerski’s study [22] suggests that women exposed to DVA and RC might use EC instead of regular contraception.

The two studies that did not find an association between DVA and EC use had some important differences from those studies that did report an association. The Estonian study [24] reported unadjusted ORs and only looked at EC use following the most recent intercourse. Such measure of the outcome at a single time point is likely to underestimate actual EC use over a 12-month timeframe of exposure. Although authors reported socio-demographic and sexual health characteristics of the sample, they did not adjust for these when assessing the association between DVA and EC use. This could result in biased estimate of the association. The Indian study [26] based on a highly selective sample provided all women with an advance supply of EC which made it easier to use it whether or not they were exposed to DVA. The improved access to EC across the sample could have impact on the actual association.

In addition, the association between DVA and EC use can be influenced by wider contextual factors such as awareness about EC among women and health-care providers, cultural and religious beliefs and attitudes, and acceptability and accessibility of EC and other methods of birth control [30, 31]. Accessibility depends on which services provide EC (e.g., pharmacy, general practice, hospital) and whether women must pay. Where EC is expensive or inaccessible, women who have restricted autonomy and access to resources may be disadvantaged. In consequence, there may be no difference in EC use between abused and non-abused women, because the greater need of the former is attenuated by poorer access. The four studies supporting the association between DVA and use of EC were conducted in USA and Nicaragua where abortion services are less acceptable and accessible than hormonal EC [32, 33]. In contrast, in Estonia abortion services are widely available and acceptable to women with unintended pregnancy [28]. In India, EC awareness and acceptability among women and health care providers are rather low [34–36].

### Limitations of the evidence base

We only found a small number of eligible primary studies, all of which had methodological limitations. Five out of six studies were cross-sectional surveys most of which did not control for the core DVA and EC covariates and mediator. Unmeasured confounders (e.g., socio-cultural norms regarding EC, accessibility of EC) could result in biased effect estimates. Although response rates for completion of questionnaires recording DVA and EC were generally high, there was a potential for differences between responders and non-responders which was not investigated by any of the studies. All studies relied on retrospective recall of the exposure and outcome which is likely to have led to either an underestimate or overestimate of the association between DVA and EC use. Self-report of DVA in all studies is likely to have resulted in under-reporting due to the stigma and social desirability [37]. The gold standard for researching DVA is a face-to-face interview, conducted by a specially trained researcher in a private setting [38]. However, only two studies used this method to evaluate the exposure [23, 26]. Only two studies measured multiple types of DVA – physical, sexual, emotional [15, 23]; the rest used narrow definitions as only physical [26] or physical/ sexual [22, 25, 28]. Not measuring emotional, psychological and financial types of DVA can result in general under-reporting of the exposure. None of the studies specifically asked about the use of the copper intrauterine device as a form of EC. While four studies specifically asked about EC pills, two did not clarify which EC method(s) they assessed [15, 22]. It is therefore possible that women in the latter studies reported use of both methods. If this is the case, EC use may have been under-reported in the four studies which asked women about EC pills.

### Strengths and limitations of the review

This is the first review to synthesise quantitative evidence of the association between exposure to DVA and use of EC. The review protocol was pre-registered in the publicly available database to ensure transparency. We used a comprehensive, sensitive search strategy across multiple databases including sources of grey literature, followed by hand searches of references and citations and contact with experts. This comprehensive approach means that it is unlikely that we have missed relevant studies. However, a formal assessment of the potential for publication bias was not possible due to the small number of included studies. We took steps throughout the review process to reduce the potential for bias and errors. Two reviewers independently screened titles and abstracts and assessed full text studies for inclusion. Data extraction and risk of bias assessment were performed by one reviewer and checked in detail by a second, and a validated tool was used to assess study quality. The small number of studies, and the different ways in which DVA and EC use were measured, meant that it was not appropriate to conduct a meta-analysis.

## Conclusion

This systematic review of six studies with 15,297 women provides some evidence of increased use of EC among younger women exposed to DVA in high- and middle-income countries. Our findings are relevant to healthcare practitioners, policy makers and commissioners involved in provision of EC. Women exposed to DVA need easy access to EC, which can be provided via different services free of charge. Request for EC can indicate possible exposure to DVA. Therefore, each consultation for EC could be an appropriate context for clinical enquiry about DVA and signposting/referral to specialist DVA services. Healthcare settings where EC is provided are suitable for the safe display of information about DVA, its impact on health, and local DVA services. DVA interventions for health-care practitioners should be targeted at core providers of EC and include new evidence on an association between exposure to DVA and use of EC.

Future studies on women’s health should use a longitudinal design and embed validated measures of DVA and RC alongside other factors associated with women’s health, and EC use alongside other reproductive health outcomes.

## Additional files

Additional file 1: PRISMA checklist. The checklist shows compliance with the PRISMA statement for preferred reporting items for systematic reviews and meta-analyses. (DOC 64 kb)

Additional file 2: Search strategy. The document describes the search strategy across electronic databases and search engines. (DOCX 18 kb)

Additional file 3: List of excluded studies with reasons for exclusion. The document lists all studies excluded at full-text screening stage categorised by reasons for exclusion. (DOCX 20 kb)

Additional file 4: Characteristics of included studies. The table provides detailed characteristics of the included studies in chronological order. (DOCX 35 kb)

## Abbreviations

DVA: Domestic violence and abuse
EC: Emergency contraception
NICE: The UK National Institute for Health and Care Excellence
NOS: Newcastle-Ottawa Scale
RC: Reproductive coercion
WHO: World Health Organisation

## References

[1] Responding to intimate partner violence and sexual violence against women: WHO clinical and policy guidelines [http://www.who.int/reproductivehealth/publications/violence/9789241548595/en/]. 24354041

[2] GOV.UK: Domestic violence and abuse. Guidance. In. Home. Office 2013. https://www.gov.uk/guidance/domestic-violence-and-abuse#domestic-violence-and-abuse-new-definition:. Accessed 23 Sept 2018.

[3] García-Moreno C (2013). Global and regional estimates of violence against women: prevalence and health effects of intimate partner violence and non-partner sexual violence.

[4] NICE (2014). Domestic violence and abuse: how social care, health services and those they work with can respond effectively.

[5] WHO (2013). Responding to Intimate Partner Violence and Sexual Violence Against Women: WHO Clinical and Policy Guidelines.

[6] WHO Reproductive Health Library. WHO recommendation on the method for clinical diagnosis of intimate partner violence in pregnancy. Geneva: The WHO Reproductive Health Library; World Health Organization; 2016. https://extranet.who.int/rhl/topics/preconception-pregnancy-childbirth-and-postpartum-care/antenatal-care/who-recommendation-clinical-diagnosis-intimate-partner-violence-pregnancy. Accessed 23 Sept 2018.

[7] Sarkar NN (2008). The impact of intimate partner violence on women's reproductive health and pregnancy outcome. J Obstet Gynaecol.

[8] Hall M, Chappell LC, Parnell BL, Seed PT, Bewley S (2014). Associations between intimate partner violence and termination of pregnancy: a systematic review and meta-analysis. PLoS Med.

[9] Clark LE, Allen RH, Goyal V, Raker C, Gottlieb AS (2014). Reproductive coercion and co-occurring intimate partner violence in obstetrics and gynecology patients. Am J Obstet Gynecol.

[10] Grace KT, Anderson JC (2016). Reproductive coercion: a systematic review. Trauma Violence Abuse Rev J.

[11] Maxwell L, Devries K, Zionts D, Alhusen JL, Campbell J (2015). Estimating the effect of intimate partner violence on women’s use of contraception: a systematic review and meta-analysis. PLoS ONE.

[12] Bergmann JN, Stockman JK (2015). How does intimate partner violence affect condom and oral contraceptive use in the United States?: a systematic review of the literature. Contraception.

[13] Cleland K, Raymond EG, Westley E, Trussell J (2014). Emergency contraception review: evidence-based recommendations for clinicians. Clin Obstet Gynecol.

[14] Cheng L, Che Y, Gulmezoglu AM (2012). Interventions for emergency contraception. Cochrane Database Syst Rev.

[15] Fantasia HC, Sutherland MA, Fontenot HB, Lee-St John TJ (2012). Chronicity of partner violence, contraceptive patterns and pregnancy risk. Contraception.

[16] CRD: Systematic reviews: CRD’s guidance for undertaking reviews in health care. In. Edited by Dissemination CfRa. Centre for Reviews and Dissemination: University of York; 2009: 294.

[17] Higgins J, Altman D, JAC S. Chapter 8: Assessing risk of bias in included studies. In: JPT H, S G, editors. Cochrane Handbook for Systematic Reviews of interventions Version 51 [updated March 2011]. The Cochrane Library, The Cochrane Collaboration (2011). Available from www.cochrane-handbook.org. The Cochrane Library: The Cochrane Collaboration 2011. http://handbook-5-1.cochrane.org/. Accessed 23 Sept 2018.

[18] Moher D, Liberati A, Tetzlaff J, Altman DG, Group P (2009). Preferred reporting items for systematic reviews and meta-analyses: the PRISMA statement. PLoS Med.

[19] The Newcastle-Ottawa Scale (NOS) for assessing the quality of nonrandomised studies in meta-analyses [http://www.ohri.ca/programs/clinical_epidemiology/oxford.htm].

[20] WHO (2006). Reproductive health indicators : guidelines for their generation, interpretation and analysis for global monitoring.

[21] Juni P, Witschi A, Bloch R, Egger M (1999). The hazards of scoring the quality of clinical trials for meta-analysis. Jama.

[22] Kazmerski T, McCauley HL, Jones K, Borrero S, Silverman JG, Decker MR, Tancredi D, Miller E (2015). Use of reproductive and sexual health services among female family planning clinic clients exposed to partner violence and reproductive coercion. Matern Child Health J.

[23] Salazar M, Ohman A (2014). Who is using the morning-after pill? Inequalities in emergency contraception use among ever partnered Nicaraguan women; findings from a national survey. Intern.

[24] Laanpere M, Ringmets I, Part K, Karro H (2013). Intimate partner violence and sexual health outcomes: a population-based study among 16-44-year-old women in Estonia. Eur J Pub Health.

[25] Gee RE, Mitra N, Wan F, Chavkin DE, Long JA (2009). Power over parity: intimate partner violence and issues of fertility control. Am J Obstet Gynecol.

[26] Rocca CH, Shankar M, Sreevathsa A, Krishnan S (2013). Acceptability and use of emergency contraception among married women in Bangalore, India. Int J Gynecol Obstet.

[27] Part K, Laanpere M, Haldre K, Rahu M, Karro H. Estonian women's health: sexual and reproductive health, health behavior, attitudes and use of health care services: survey report. Tartu: Tartu Ülikooli Naistekliinik; 2007. Accessed 23 Sept 2018.

[28] Laanpere M, Ringmets I, Part K, Karro H (2010). Violence and fertility control: results from the survey among 16-44-year old women in Estonia. J Psychosom Obstet Gynecol.

[29] Miller E, Jordan B, Levenson R, Silverman JG (2010). Reproductive coercion: connecting the dots between partner violence and unintended pregnancy. Contraception.

[30] Westley E, Kapp N, Palermo T, Bleck J (2013). A review of global access to emergency contraception. Int J Gynaecol Obstet.

[31] Westley E, Schwarz EB (2012). Emergency contraception: global challenges, new opportunities. Contraception.

[32] Federal and state bans and restrictions on abortion. In*.* https://www.plannedparenthoodaction.org/issues/abortion/federal-and-state-bans-and-restrictions-abortion: Planned Parenthood Action Fund.

[33] ICEC: ICEC worldwide case studies: Latin America. Building regional alliances to promote emergency contraception. In*.* http://www.cecinfo.org/publications-and-resources/icec-publications/#pub8: International Consortium for emergency contrcaption.

[34] ICEC: Counting what counts: tracking access to emergency contraception. In: Revitalizing the emergency contraception agenda. http://www.cecinfo.org/publications-and-resources/icec-publications/#pub8: International Consortium for Emergency Contraception; 2013.

[35] Kumar M, Meena J, Sharma S, Poddar A, Dhalliwal V, Modi-Satish Chander Modi SC, Singh K (2011). Contraceptive use among low-income urban married women in India. J Sex Med.

[36] Prateek SS, Saurabh RS (2012). Contraceptive practices adopted by women attending an urban health Centre. Afr Health Sci.

[37] Ruiz-Perez I, Plazaola-Castano J, Vives-Cases C (2007). Methodological issues in the study of violence against women. J Epidemiol Community Health.

[38] WHO (2001). Putting women first: ethical and safety recommendations for research and domestic violence against women.

