# Supplementary material for: Use of emergency contraception among women with experience of domestic violence and abuse: a systematic review
Source: BMC Womens Health. 2018 Sep 25;18:156. doi: 10.1186/s12905-018-0652-7 (PMC6156954; doi:10.1186/s12905-018-0652-7)
Supplement: Supplementary file 2 — Search strategy. The document describes the search strategy across electronic databases and search engines. (DOCX 18 kb) [file 12905_2018_652_MOESM2_ESM.docx]

**Additional file 2.** Search strategy

**Electronic searching**

Date of search October 7^th^ 2016, updated December 2017.

| Search results | Medline/Premedline = 497  Embase= 675  Cochrane = 24  Cinahl=109  Psycinfo= 179  Web of Science= 428  Total= 1912  Total deduplicated = 1088 |
| --- | --- |
|  | All languages |

**MEDLINE**

Database: Epub Ahead of Print, In-Process & Other Non-Indexed Citations, Ovid MEDLINE(R) Daily and Ovid MEDLINE(R) <1946 to Present>

Search Strategy:

--------------------------------------------------------------------------------

1 domestic violence/ or exp intimate partner violence/ or battered women/ (12823)

2 (rape/ or sex offenses/ or violence/ or physical abuse/ or coercion/ or crime victims/) and (spouses/ or marriage/ or Sexual partners/) (1543)

3 (rape/ or sex offenses/ or violence/ or physical abuse/ or coercion/ or crime victims/) and (domestic or spous* or partner* or married or marriage* or marital or husband* or wife or wives or boyfriend* or girlfriend*).tw. (4600)

4 ((domestic or spous* or partner* or married or marriage* or marital or husband* or wife or wives or boyfriend* or girlfriend*) adj4 (abus* or victim* or violent or violence or assault* or beat or beating or batter* or rape* or sex offense* or sexual offense*)).tw. (12750)

5 (spouses/ or marriage/ or Sexual partners/) and (abus* or victim* or violent or violence or assault* or beat or beating or batter* or rape* or sex offense* or sexual offense*).tw. (3501)

6 (IPV or DVA).tw. (4683)

7 (battered adj2 (woman or women)).tw. (630)

8 ((woman or women) adj3 relationship* adj3 abus*).tw. (86)

9 ((birth control or fertility control or reproductiv* or contraceptiv* or contraception or sexual) adj3 (sabotag* or coerc*)).tw. (718)

10 or/1-9 (22202)

11 Contraception, Postcoital/ or exp Contraceptives, Postcoital/ (8576)

12 ((emergency or postcoital or fail* or "use" or nonuse or non-use) adj3 (contraceptiv* or contraception or birth control or fertility control)).tw. (18788)

13 *contraception/ or Contraception Behavior/ or *contraceptive agents/ or *contraceptive agents, female/ or *contraceptives, oral/ (33258)

14 morning after pill*.tw. (206)

15 "Levonorgestrel"/ (3801)

16 (levonorgestrel or ulipristal or levonelle or ellaone).tw. (4212)

17 (yuzpe adj2 (regim* or method*)).tw. (137)

18 Pregnancy, Unplanned/ or pregnancy, unwanted/ (3525)

19 ((unplann* or unwant* or unintend*) adj3 pregnan*).tw. (6945)

20 ((reproductiv* or contraceptiv* or contraception) adj3 control*).tw. (2287)

21 ((pharmacies or pharmacy or pharmacist*) adj3 (contraceptiv* or contraception)).tw. (116)

22 or/11-21 (59355)

23 10 and 22 (505)

24 letter/ (943752)

25 editorial/ (420550)

26 news/ (180640)

27 exp historical article/ (382031)

28 Anecdotes as topic/ (4740)

29 comment/ (686911)

30 (letter or comment$).ti. (118881)

31 animals/ not humans/ (4292843)

32 exp Animals, Laboratory/ (791688)

33 exp Animal Experimentation/ (8220)

34 exp Models, Animal/ (482912)

35 exp rodentia/ (2937932)

36 (rat or rats or mouse or mice).ti. (1223396)

37 or/24-36 (7111929)

38 23 not 37 (497)

**Grey literature search**

Date of search: 9^th^ November 2016.

Key words: domestic violence, domestic abuse, DVA, intimate partner violence, IPV, emergency contraception, morning after pill.

Sources:

Google (first 6 pages), NICE, NHS Choices and Department of Health websites for policy and guidance documents, Clinical trial registers, websites of relevant medical and pharmacy associations and charities in the field of reproductive health and DVA.
