# Supplementary material for: Use of emergency contraception among women with experience of domestic violence and abuse: a systematic review
Source: BMC Womens Health. 2018 Sep 25;18:156. doi: 10.1186/s12905-018-0652-7 (PMC6156954; doi:10.1186/s12905-018-0652-7)
Supplement: Supplementary file 3 — List of excluded studies with reasons for exclusion. The document lists all studies excluded at full-text screening stage categorised by reasons for exclusion. (DOCX 20 kb) [file 12905_2018_652_MOESM3_ESM.docx]

**Additional file 3**

List of excluded studies with reasons for exclusion (n=90)

**Commentary (n=1)**

Kurniati A, Chen CM, Efendi F, Berliana SM. Factors influencing Indonesian women's use of maternal health care services. *Health Care Women Int* 2017.

**No comparative group of women unexposed to DVA (n=1)**

Pham A, Ryan N, Joscelyne A, Keller AS, Ades V. Gynecologic needs among a population of survivors of torture in New York City. *Annals of Global Health* 2016;**82**:552.

**No exposure to DVA (n=5)**

Morhason-Bello IO, Adedokun BO, Mumuni TO, Bello FA, Abdus-Salam RA, Lawal OO*, et al.* Knowledge and use of emergency contraception by medical doctors on internship in a tertiary healthcare facility in Nigeria. *Niger J Clin Pract* 2014;**17**:431-5.

Wilder KJ, Guise JM, Perrin NA, Hanson GC, Hernandez R, Glass N. Knowledge, Awareness, Perceptions, and Use of Emergency Contraceptives among Survivors of Intimate Partner Violence.

Abate M, Assefa N, Alemayehu T. Knowledge, attitude, practice, and determinants emergency contraceptive use among women seeking abortion services in dire dawa, ethiopia. *PLoS ONE* 2014;**9 (10) (no pagination)**.

Samari G. Women's empowerment and short- and long-acting contraceptive method use in Egypt. *Culture, Health & Sexuality* 2017;

Okigbo CC. A multilevel analysis of socioecological factors associated with modern contraceptive use in urban Nigeria. *Dissertation Abstracts International: Section B: The Sciences and Engineering* 2017;**77**.

**Systematic review (n=5)**

Salam RA, Faqqah A, Sajjad N, Lassi ZS, Das JK, Kaufman M*, et al.* Improving Adolescent Sexual and Reproductive Health: A Systematic Review of Potential Interventions. *Journal of Adolescent Health* 2016;**59**:S11-S28.

Maxwell L, Devries K, Zionts D, Alhusen JL, Campbell J. Estimating the effect of intimate partner violence on women's use of contraception: a systematic review and meta-analysis. *PLoS ONE [Electronic Resource]* 2015;**10**:e0118234.

Mmari K, Sabherwal S. A review of risk and protective factors for adolescent sexual and reproductive health in developing countries: an update. *Journal of Adolescent Health* 2013;**53**:562-72.

Coker AL. Does physical intimate partner violence affect sexual health? A systematic review. *Trauma Violence Abuse Rev J* 2007;**8**:149-77.

Holliday CN. Racial/ethnic disparities in unintended pregnancy, reproductive coercion, and intimate partner violence. *Dissertation Abstracts International: Section B: The Sciences and Engineering* 2015;**76**:No Pagination Specified.

Martinez-Leon NC, Pena JJ, Salazar H, Garcia A, Sierra JC. A systematic review of romantic jealousy in relationships. *Terapia Psicologica* 2017;**35**:203-12.

**Measure of EC and DVA but no analysis (n=7)**

Pack AP, McCarraher DR, Chen M, Okigbo CC, Albert LM, Wambugu S. Factors associated with unmet need for modern contraception in post-conflict Liberia. *Afr J Reprod Health* 2014;**18**:58-67.

Williams CM, Larsen U, McCloskey LA. Intimate partner violence and women's contraceptive use. *Violence Against Women* 2008;**14**:1382-96.

Laanpere M, Ringmets I, Part K, Karro H. Violence and fertility control: Results from the survey among 16-44-year old women in Estonia. *Journal of Psychosomatic Obstetrics and Gynecology* 2010;**31**:112. http://dx.doi.org/http://dx.doi.org/10.3109/0167482x.2010.536387

Nelson DB, Zhao H, Corrado R, Mastrogiannnis DM, Lepore SJ. Preventing Unintended Pregnancy Among Young Sexually Active Women: Recognizing the Role of Violence, Self-Esteem, and Depressive Symptoms on Use of Contraception. *Journal of Women's Health* 2017;**26**:352-60. http://dx.doi.org/https://dx.doi.org/10.1089/jwh.2016.5753

Dasgupta A. Understanding intimate partner violence and associated challenges to family planning among married women in Maharashtra, India. *Dissertation Abstracts International: Section B: The Sciences and Engineering* 2016;**77**.

Jones KA. Negative male partner influences on reproductive health and contraceptive use among adolescent and young adult women. *Dissertation Abstracts International: Section B: The Sciences and Engineering* 2017;**77**.

Miller E, Decker MR, Raj A, Reed E, Marable D, Silverman JG. Intimate Partner Violence and Health Care-Seeking Patterns Among Female Users of Urban Adolescent Clinics. *Maternal and Child Health Journal* 2010;**14**:910-7.

**No Measure of contraception (n=13)**

Abused women are more likely to rely on condoms than birth control pills to prevent pregnancies. *AHRQ Research Activities* 2009, 1.

Colarossi L, Dean G. Partner violence and abortion characteristics. *Women Health* 2014;**54**:177-93.

Holliday CN, McCauley HL, Silverman JG, Ricci E, Decker MR, Tancredi DJ*, et al.* Racial/Ethnic Differences in Women's Experiences of Reproductive Coercion, Intimate Partner Violence, and Unintended Pregnancy. *Journal of Women's Health* 2017;**26**:828-35.

Hovsepian SL, Blais M, Manseau H, Otis J, Girard ME. Prior victimization and sexual and contraceptive self-efficacy among adolescent females under Child Protective Services care. *Health Educ Behav* 2010;**37**:65-83.

McCleary-Sills JD. Intimate partner violence and women's reproductive agency in Jordan. *Dissertation Abstracts International: Section B: The Sciences and Engineering* 2012;**72**:5922.

Meiksin R, Meekers D, Thompson S, Hagopian A, Mercer MA. Domestic violence, marital control, and family planning, maternal, and birth outcomes in Timor-Leste. *Matern Child Health J* 2015;**19**:1338-47.

Miller E, Decker M, McCauley H, Levenson R, Tancredi D, Silverman J. Partner violence, reproductive control, and pregnancy experiences among adolescent female users of family planning clinics. *Journal of Adolescent Health* 2010;**1)**:S32-S3.

Miller E, Decker MR, McCauley HL, Tancredi DJ, Levenson RR, Waldman J*, et al.* Pregnancy coercion, intimate partner violence and unintended pregnancy. *Contraception* 2010;**81**:316-22. http://dx.doi.org/10.1016/j.contraception.2009.12.004

Miller E, McCauley HL, Tancredi DJ, Decker MR, Anderson H, Silverman JG. Recent reproductive coercion and unintended pregnancy among female family planning clients. *Contraception* 2014;**89**:122-8.

Oberg M, Stenson K, Skalkidou A, Heimer G. Prevalence of intimate partner violence among women seeking termination of pregnancy compared to women seeking contraceptive counseling. *Acta Obstetricia et Gynecologica Scandinavica* 2014;**93**:45-51.

Stockl H, Hertlein L, Himsl I, Delius M, Hasbargen U, Friese K*, et al.* Intimate partner violence and its association with pregnancy loss and pregnancy planning. *Acta Obstetricia et Gynecologica Scandinavica* 2012;**91**:128-33.

Valencia A. A comparative analysis of intimate partner violence using an ecological framework. *Dissertation Abstracts International Section A: Humanities and Social Sciences* 2012;**72**:2585.

Zakar R, Zakar MZ, Mikolajczyk R, Kramer A. Intimate partner violence and its association with women's reproductive health in Pakistan. *Int J Gynaecol Obstet* 2012;**117**:10-4.

**Contraception but no measure of EC (n=57)**

What impact does intimate partner violence have on reproductive decision-making? *Contraceptive Technology Update* 2015;**36**:115-6.

Adjiwanou V, N'Bouke A. Exploring the Paradox of Intimate Partner Violence and Increased Contraceptive Use in sub-Saharan Africa. *Stud Fam Plann* 2015;**46**:127-42.

Agenor M, Austin SB, Kort D, Austin EL, Muzny CA. Sexual Orientation and Sexual and Reproductive Health among African American Sexual Minority Women in the U.S. South. *Womens Health Iss* 2016;**26**:612-21.

Alio AP, Daley EM, Nana PN, Duan J, Salihu HM. Intimate partner violence and contraception use among women in Sub-Saharan Africa. *Int J Gynaecol Obstet* 2009;**107**:35-8.

Allsworth JE, Secura GM, Zhao Q, Madden T, Peipert JF. The impact of emotional, physical, and sexual abuse on contraceptive method selection and discontinuation. *Am J Public Health* 2013;**103**:1857-64.

Anderson JC, Grace KT, Miller E. Reproductive coercion among women living with HIV: an unexplored risk factor for negative sexual and mental health outcomes. *Aids* 2017;**31**:2261-5.

Antai D, Adaji S. Community-level influences on women's experience of intimate partner violence and terminated pregnancy in Nigeria: a multilevel analysis. *BMC Pregnancy Childbirth* 2012;**12**:128. Azevedo AC, Araujo TV, Valongueiro S, Ludermir AB. Intimate partner violence and unintended pregnancy: prevalence and associated factors. *Cad Saude Publica* 2013;**29**:2394-404.

Beadnell B, Baker SA, Morrison DM, Knox K. HIV/STD Risk Factors for Women with Violent Male Partners. *Sex Roles* 2000;**42**:661-89.

Blom H, Hogberg U, Olofsson N, Danielsson I. Violence victimisation associated with sexual ill health and sexual risk behaviours in Swedish youth. *International Journal of Gynecology and Obstetrics* 2015;**131**:E80.

Boyce SC, McDougal L, Silverman JG, Atmavilas Y, Dhar D, Hay K*, et al.* Associations of intimate partner violence with postnatal health practices in Bihar, India. *BMC Pregnancy Childbirth* 2017;**17**:398. http://dx.doi.org/https://dx.doi.org/10.1186/s12884-017-1577-0

Cha S, Chapman DA, Wan W, Burton CW, Masho SW. Intimate partner violence and postpartum contraceptive use: the role of race/ethnicity and prenatal birth control counseling. *Contraception* 2015;**92**:268-75.

Chacham AS, Simao AB, Caetano AJ. Gender-based violence and sexual and reproductive health among low-income youth in three Brazilian cities. *Reproductive Health Matters* 2016;**24**:141-52.

Chan RL, Martin SL. Physical and sexual violence and subsequent contraception use among reproductive aged women. *Contraception* 2009;**80**:276-81.

Dalal K, Andrews J, Dawad S. Contraception use and associations with intimate partner violence among women in Bangladesh. *Journal of Biosocial Science* 2012;**44**:83-94.

Dasgupta A, Saggurti N, Donta B, Battala M, Ghule M, Nair S*, et al.* Intimate partner violence and condom versus other modern contraception use among married women in rural India. *Annals of Global Health* 2015;**81 (1)**:218-9.

DiLernia J. The effect of domestic violence on contraceptive use among adolescents in nicaragua. *Contraception* 2014;**90 (3)**:314.

Diop-Sidibe N, Campbell JC, Becker S. Domestic violence against women in Egypt--wife beating and health outcomes. *Soc Sci Med* 2006;**62**:1260-77.

Early D, Cross Riedel J, Thiel De Bocanegra H, Swann D, Schwarz EB. Differences in contraceptive use between women reporting reproductive coercion and intimate partner violence. *Contraception* 2015;**92 (4)**:362.

Fanslow J, Whitehead A, Silva M, Robinson E. Contraceptive use and associations with intimate partner violence among a population-based sample of New Zealand women. *Aust N Z J Obstet Gynaecol* 2008;**48**:83-9.

Forrest W, Arunachalam D, Navaneetham K. Intimate Partner Violence and Contraceptive Use in India: The Moderating Influence of Conflicting Fertility Preferences and Contraceptive Intentions. *Journal of Biosocial Science* 2017;

Garoma S, Belachew T, Wondafrash M. Sexual coercion and reproductive health outcomes among young females of Nekemte Town, South West Ethiopia. *Ethiop Med J* 2008;**46**:19-28.

Gogoi M, Kumar A. Socioeconomic counters of intimate partner violence and impact on reproductive health services in India. *Tropical Medicine and International Health* 2017;**22**:101-2.

Gomez AM. Sexual violence as a predictor of unintended pregnancy, contraceptive use, and unmet need among female youth in Colombia. *Journal of Women's Health* 2011;**20**:1349-56.

Goossens J, Van Den Branden Y, Van der Sluys L, Delbaere I, Van Hecke A, Verhaeghe S*, et al.* The prevalence of unplanned pregnancy ending in birth, associated factors, and health outcomes. *Hum Reprod* 2016;**31**:2821-33.

Hall KS, Harris LH, Dalton VK. Women's Preferred Sources for Primary and Mental Health Care: Implications for Reproductive Health Providers. *Womens Health Iss* 2017;**27**:196-205.

Hathaway JE, Mucci LA, Silverman JG, Brooks DR, Mathews R, Pavlos CA. Health status and health care use of Massachusetts women reporting partner abuse. *Am J Prev Med* 2000;**19**:302-7.

Hoang A, Nguyen CQ, Duong CD. Youth experiences in accessing sexual healthcare services in Vietnam. *Culture, Health & Sexuality* 2017;

Huneeus A, Fernandez M, Parra P, Zakharova A, Schilling A. Gender differences on adolescent sexual behavior. *Journal of Pediatric and Adolescent Gynecology* 2017;**30**:305.

Kojo T, Ae R, Tsuboi S, Nakamura Y, Kitamura K. Differentials in variables associated with past history of artificial abortion and current contraception by age: Results of a randomized national survey in Japan. *Journal of Obstetrics and Gynaecology Research* 2017;**43**:516-22.

Kusunoki Y, Barber J, Gatny H, Melendez R. Intimate partner violence and contraceptive behaviors among young women. *Contraception* 2014;**90 (3)**:295.

Kusunoki Y, Barber JS, Gatny HH, Melendez R. Physical Intimate Partner Violence and Contraceptive Behaviors Among Young Women. *Journal of Women's Health* 2017;**28**:28.

Mahapatro M, Gupta RN, Gupta V, Kundu AS. Interpersonal violence as risk factor for women's sexually transmitted infection and reproductive health consequences in India: A community based study. *Journal of Public Health (Germany)* 2012;**20**:399-403.

Manlove J, Ryan S, Franzetta K. Contraceptive use and consistency in U.S. teenagers' most recent sexual relationships. *Perspect Sex Reprod Health* 2004;**36**:265-75.

Miller MK, Randell KA, Barral R, Sherman AK, Miller E. Factors Associated With Interest in Same-Day Contraception Initiation Among Females in the Pediatric Emergency Department. *Journal of Adolescent Health* 2016;**58**:154-9.

Mody SK, Nair S, Dasgupta A, Raj A, Donta B, Saggurti N*, et al.* Postpartum contraception utilization among low-income women seeking immunization for infants in Mumbai, India. *Contraception* 2014;**89**:516-20.

Mundhra R, Singh N, Kaushik S, Mendiratta A. Intimate Partner Violence: Associated Factors and Acceptability of Contraception Among the Women. *Indian J* 2016;**41**:203-7.

Murshid NS. Intimate partner violence and contraception in Pakistan: Results from Pakistan Demographic and Health Survey 2012-13. *Women Stud Int Forum* 2017;**64**:10-6.

Murshid NS, Ely GE. Intimate partner violence and use of contraceptives in Bangladesh: Results from a national sample. *International Journal of Social Welfare* 2016;**25**:331-8.

Nguyen PH, Nguyen SV, Nguyen MQ, Nguyen NT, Keithly SC, Mai LT*, et al.* The association and a potential pathway between gender-based violence and induced abortion in Thai Nguyen province, Vietnam. *Glob Health Action* 2012;**5**:1-11.

Northridge JL, Silver EJ, Talib HJ, Coupey SM. Reproductive Coercion in High School-Aged Girls: Associations with Reproductive Health Risk and Intimate Partner Violence. *J Pediatr Adolesc Gynecol* 2017;**30**:603-8.

O'Hara K, Tsai LC, Carlson CE, Haidar YM. Experiences of intimate-partner violence and contraception use among ever-married women in Jordan. *East Mediterr Health J* 2013;**19**:876-82.

Okenwa L, Lawoko S, Jansson B. Contraception, reproductive health and pregnancy outcomes among women exposed to intimate partner violence in Nigeria. *Eur J Contracept Reprod Health Care* 2011;**16**:18-25.

Raj A, McDougal L, Reed E, Silverman JG. Associations of marital violence with different forms of contraception: cross-sectional findings from South Asia. *Int J Gynaecol Obstet* 2015;**130 Suppl 3**:E56-61.

Rickert VI, Wiemann CM, Harrykissoon SD, Berenson AB, Kolb E. The relationship among demographics, reproductive characteristics, and intimate partner violence. *Am J Obstet Gynecol* 2002;**187**:1002-7.

Roberts TA, Auinger P, Klein JD. Intimate partner abuse and the reproductive health of sexually active female adolescents. *Journal of Adolescent Health* 2005;**36**:380-5.

Rosenfeld EA, Miller E, Zhao X, Sileanu FE, Mor MK, Borrero S. Male partner reproductive coercion among women veterans. *Am J Obstet Gynecol* 2017;**19**:19.

Salazar M, Valladares E, Hogberg U. Questions about intimate partner violence should be part of contraceptive counselling: findings from a community-based longitudinal study in Nicaragua. *J Fam Plann Reprod Health Care* 2012;**38**:221-8.

Silverman JG, Raj A, Mucci LA, Hathaway JE. Dating violence against adolescent girls and associated substance use, unhealthy weight control, sexual risk behavior, pregnancy, and suicidality. *Jama* 2001;**286**:572-9.

Singh N, Shukla SK. Does violence affect the use of contraception? Identifying the hidden factors from rural India. *Journal of Family Medicine & Primary Care* 2017;**6**:73-7.

Stephenson R, Jadhav A, Hindin M. Physical domestic violence and subsequent contraceptive adoption among women in rural India. *Journal of Interpersonal Violence* 2013;**28**:1020-39.

Stephenson R, Koenig MA, Acharya R, Roy TK. Domestic violence, contraceptive use, and unwanted pregnancy in rural India. *Stud Fam Plann* 2008;**39**:177-86.

Stockman J, Campbell J, Campbell D, Sharps P, Callwood G. Sexual intimate partner violence, sexual risk behaviors, and contraceptive practices among women of African descent. *Contraception* 2010;**82 (2)**:212.

Tsai LC, Cappa C, Petrowski N. The Relationship between Intimate Partner Violence and Family Planning among Girls and Young Women in the Philippines. *Glob J Health Sci* 2016;**8**:54382.

Viswan SP, Ravindran TKS, Kandala NB, Petzold MG, Fonn S. Sexual autonomy and contraceptive use among women in Nigeria: findings from the Demographic and Health Survey data. *Int J Women Health* 2017;**9**:581-90.

Wingood GM, DiClemente RJ. The effects of an abusive primary partner on the condom use and sexual negotiation practices of African-American women. *Am J Public Health* 1997;**87**:1016-8.
