# Supplementary material for: Use of emergency contraception among women with experience of domestic violence and abuse: a systematic review
Source: BMC Womens Health. 2018 Sep 25;18:156. doi: 10.1186/s12905-018-0652-7 (PMC6156954; doi:10.1186/s12905-018-0652-7)
Supplement: Supplementary file 4 — Characteristics of included studies. The table provides detailed characteristics of the included studies in chronological order. (DOCX 35 kb) [file 12905_2018_652_MOESM4_ESM.docx]

**Additional file 4.**

**Table. Characteristics of included studies (in chronological order)**

| **First author, year, country** | **Study design**  **Setting** | **Study sample** | **Data collection method** | **Measure of exposure** | **DVA prevalence** | **Measure of outcome** | **EC use prevalence** |
| --- | --- | --- | --- | --- | --- | --- | --- |
| Fantasia 2012 [15], northeastern USA | Cross sectional  Four reproductive health clinics | N=2000  Age: Mean 21.9 years (SD 6.5)  Race/Ethnicity:  White n=1297; 64.9%, Asian 45; 2.3%  Black n=114; 5.7%  American Indian/ Alaskan Native 12; 0.6%  Don’t know/ refused 462; 23.1%  Other 49; 2.5%  Multiracial 21; 1%  Ethnicity  Non-Hispanic/Latino 1261; 63.1%  Hispanic/Latino 599; 30%  Arabic 2; 0.1%  Not reported 138; 6.9%  Relationship status:  Single n=1753; 87.7%  Married 108; 5.4%  Engaged 43; 2.2%  Separated/ widowed/divorced 52; 2.1%  Not completed 44; 2.7% | 2006-2011  Data abstracted from medical records; retrospective | Physical/psychological/sexual DVA in the:  1) past year,  2) past year and up to past 5 years,  3) past year and over more than 5 years,  4) not in the past year but there is a history before that  Self-developed | N=569; 28.5%  1) Past year 118; 5.9%  2) Past year and up to  past 5 years 55; 2.8%  3) Past year and over more than 5 years 30; 1.5%  4) Not in the past year but there is a history before that 366; 18.3% | Use of EC in the past year | Not reported |
| Gee 2013 [25], USA,  Pennsylvania | Cross-sectional  Surgical  abortion clinic and general gynecologic  clinic | N=1354  Age:  18-24 years n=132; 45.4%  25-34 years n=136; 46.7%  35-44 years n=22; 7.6%  45-54 years n=1; 0.3%  Ethnicity:  Black n=331 (24%)  White n= 834 (60%) Other n=222 (16%) | 2007  Self-administered paper questionnaire; retrospective | Lifetime physical/psychological/sexual DVA  DVA module from the CDC’s Behavioural Risk Factor Surveillance System (BRFSS) [39] | n=291; 21.0%  Husband: exposed to DVA n=16;5.5%  Not exposed to DVA n=85; 7.8%  Boyfriend: exposed to DVA n=44; 15.1% Not exposed to DVA n=208; 19.0% | Lifetime use of EC | n = 404; 37% |
| Laanpere 2013 [24], Estonia | National cross-sectional  Households | N=1966  Age:  16-24 years n=755  25-34 years n=585  35-44 years n=626  Ethnicity:  Estonian n=1391  Non-Estonian n=570  Unknown n=5  Relationship status:  Married: 1280; 65%  Divorced/separated 189; 9.6%  Single 493; 25% | 2004-2005  Self-administered postal questionnaire; retrospective | Physical/sexual DVA in the past year  Self-developed | n=362; 18.4%  16-24 years n=155; 20.5%  25-25 n=112; 20.9%  35-44 n=26.2; 15.2%  Physical DVA 17.2%  Sexual DVA 4.1%  Both physical and sexual 1.8% | Use of hormonal EC after the most recent sexual intercourse | n=21; 1.2% |
| Rocca 2013 [26], India, Bangalore | Prospective year-long cohort with intervention on the outcome (advance supply of EC)  Two government health centers and low-income communities | N=322  Analysed N =263  Age: Mean 22.9 SD=2.3 Range 18-25 years  Ethnicity: all Indian  Religion:  Hindu 164;50.9%  Christian 115;35.7%  Muslim 43;13.4%  Marrital status: Married n=322; 100%  Arranged marriage: 222; 68.9%  Love marriage: 100; 31.1% | 1 year (during a 2-year study in2005-2008)  Face to face interviews; retrospective | Physical DVA in the past six months  Self-developed | n=88;27.3% | Use of EC, frequency, and timing in the past year | n=37; 14.1% |
| Salazar 2014 [23], Nicaragua | National cross-sectional  Households | N=8284  Age: Mean 28.3 (SD=11.3 SE=0.13) Range = 15-49 | 2006-2007  Face to face interviews; retrospective | Lifetime emotional, physical, sexual DVA and controlling  behavior  Measure from the WHO Multi-country study[3] | Physical DVA: 23.4% (95% CI 22.3-24.6)  Emotional DVA: 43.9% (95% CI 42.4 - 45.5  Sexual DVA: 11.0% (95% 10.1– 11.9%)  Controlling behaviour by partner: 54.0% (95% CI 52.3-55.6) | Lifetime use of hormonal EC | n=383; 6% |
| Kazmerski 2015 [22],  USA, Northern California | Cross sectional  Five family planning clinics | N=1262  Age: 21.7 mean (SD 3.47) Range 16-29 years  Ethnicity: Asian 68; 5.4%  Black or African American 352; 27.9%  Hispanic 378; 30.0%  Native Hawaiian/Pacific Islander/American Indian/  Alaskan Native 72; 5.7%  White 285; 22.6%  Multiracial/ other 105; 8.5%  Relationship status:  Single: 370; 29.3%  Dating more than 1 person: 49; 3.9%  In a serious relationship:730; 57.8%  Married: 113; 9% | 2008-2009  Self-administered audio computer assisted survey; retrospective | Physical/sexual DVA and/or RC in the past three months  Self-developed from:  Modified Conflicts tactics scale-2 (CTS-2)[40]  Sexual experiences survey[41]  RC questions by Miller et al [42] | DVA only = 16.3% (95% CI 11.4–21.2, n= 206)  No DVA 83.7% (95% CI 78.8-88.6, n = 1056)  RC only = 13.5% (95% CI 10.6-16.4, n= 170)  DVA and RC =4.4% | Frequency of use of EC in the past three months (once/>once) | Not reported |

Note. DVA domestic violence and abuse. RC Reproductive coercion. EC emergency contraception.
